# Monitoring HIV Viral Load in Resource Limited Settings: Still a Matter of Debate?

**DOI:** 10.1371/journal.pone.0047391

**Published:** 2012-12-06

**Authors:** Mireia Arnedo, Elena Alonso, Nell Eisenberg, Laura Ibáñez, Cecilia Ferreyra, Angels Jaén, Laurence Flevaud, Samuel Khamadi, Paul Roddy, Jose Maria Gatell, David Dalmau, Busia OR Study Group

**Affiliations:** 1 Médécins sans Frontières-Operational Center Barcelona Athens, Barcelona, Spain; 2 Hospital Clínic de Barcelona, IDIBAPS, Barcelona, Spain; 3 Hospital Universitari MútuaTerrassa, Medicine Department, Terrassa, Spain; 4 Fundació Docència i Recerca MutuaTerrassa, Terrassa, Spain; 5 Kenya Medical Research Institute (KEMRI), Nairobi, Kenya; McGill University AIDS Centre, Canada

## Abstract

**Introduction:**

Consequences of lack of viral monitoring in predicting the effects of development of HIV drug resistance mutations during HAART in resource-limited settings (RLS) is still a matter of debate.

**Design:**

To assess, among HIV+ patients receiving their first-line HAART, prevalence of virological failure and genotypic resistance mutations pattern in a Médécins Sans Frontières/Ministry of Health programme in Busia District (Kenya).

**Methods:**

Patients with HAART treatment for ≥12 months were eligible for the study and those with HIV-RNA ≥5000 copies/ml underwent genotypic study. Total HIV-1 RNA from Dried Blood Spots was extracted using Nuclisens method.

**Results:**

926 patients were included. Among 274 (29.6%) patients with detectable viral load, 55 (5.9%) experienced treatment failure (viral load >5.000 copies/ml); 61.8% were female and 10 (18.2%) had clinical failure. Median CD4 cell count was 116 cell/mm3 (IQR: 54–189). Median HIV-RNA was 32,000 copies/ml (IQR: 11000–68000). Eighteen out of 55 (33%) samples could be sequenced on PR and RT genes, with resistance associated mutations (RAMs) in 15 out of 18 samples (83%). Among patients carrying RAMs, 12/15 (81%) harboured RAMs associated to thymidine analogues (TAMs). All of them (100%) showed M184V resistance associated mutation to lamivudine as well as NNRTI's RAMS.

**Conclusions:**

Virological failure rate in resource-limited settings are similar to those observed in developed countries. Resistance mutation patterns were concordant with HAART received by failing patients. Long term detectable viral load confers greater probability of developing resistance and as a consequence, making difficult to find out a cost-effective subsequent treatment regimen.

## Introduction

Early virological failure on a nucleoside reverse transcriptase inhibitor (NRTI) and nonnucleoside reverse transcriptase inhibitor (NNRTI) regimen is associated with emergence of the M184V mutation and an NNRTI resistance mutation in approximately 50–75% of patients in resource-rich settings [1], [2], [3].

Continuation of a failing regimen may be associated with more complex mutation patterns, as has been observed in several studies in developing countries [4], [5], [6], [7], [8]. The number and pattern of resistance mutations may depend on the exact components of the regimen, HIV-1 subtype [9], [10] and the duration of failure. Substantial NRTI resistance may occur, making empiric selection of second-line NRTI difficult.

Sub-Saharan Africa need to face major challenges to the scaling up of treatment programs such is scarcity in financial and human resources and inadequate health-care infrastructure. Models of care must be adapted to these circumstances, accounting for the few trained health personnel, different groups of patients compared with high-income countries (increased proportions of children and women of child-bearing age affected), restricted drug availability and procurement, inadequate access to monitoring equipment, and few extra funds.

Scaling up antiretroviral (ARV) therapy in RLS requires a simplified approach. Because of inadequate laboratory capacity, many programs have minimized laboratory monitoring in an effort to accelerate widespread availability of HIV treatment [11]. However, is no longer valid the topic saying that majority of HIV-infected people are still unable to access treatment and that resources should be applied to prevention measures and to the initiation of treatment, rather than to performance of expensive laboratory tests used to monitor patients who are already receiving treatment. Continuous expansion of HIV-infected people receiving ARV therapy in RLS increases the need to detect cases in which first-line treatment has failed. As the need for viral load testing increases, technologies to determine the viral load are becoming simpler, and costs are decreasing. Thus, question of whether high-quality, effective HIV care can be provided without viral load monitoring needs to be revisited.

If one of the main issues that remain still a matter of debate in low and middle income countries is the suitability for using viral load monitoring, does it make sense to use it in order to predict development of resistance mutations to HAART?

The aim of the study was to assess, among HIV+ patients receiving their first-line HAART and with virological failure (viral load >5.000 copies/ml), the prevalence of genotypic resistance mutations and their pattern. Technology used was dried blood spots filter paper (DBS), due to its simplicity and feasibility in resource limited settings.

## Methods

### Setting

Médécins sans Frontieres Spain (MSF-S) has provided ARV treatment since July 2003 in Busia District Hospital, in the western region of Kenya (Western province). Busia has a population of approximately 430,000 and HIV prevalence of 5.9% (age 15–49 years). After a period of expansion and scale-up of patient numbers, the project focused on strengthening the care and treatment of patients, having started around 3.500 patients on treatment at district level and rural level through decentralised provision of care by December 2008.

### Study population

The first-line regimen in Kenya when this study was performed consisted in stavudine (d4T), lamivudine (3TC), and nevirapine (NVP). In the event of toxicity, one can substitute zidovudine (ZDV) for d4T or efavirenz (EFV) for NVP, using TDF/3TC or abacavir (ABC)/didanosine (DDI) for patients failing first-line treatment [12].

In April 2008, all the adult ART-naïve patients receiving a triple ARV therapy regimen classified as standard first-line (e.g. D4T or AZT, 3TC and either NVP or EFV) for 12 months or more, who had attended the clinic at least once within the previous 6 months, and had given informed consent to participate, were considered for the study. The most widely ATR drug used is Triomune^R^, a fixed-dose formulation containing a combination of 2 nucleoside reverse transcriptase inhibitors (stavudine and lamivudine) with the non-nucleoside reverse transcriptase inhibitor nevirapine. Triomune can be taken twice daily, irrespective of food.

As of mid-April 2008 there were 1037 adult, active, previously treatment-naïve patients who were receiving standard fist line ARV for more than 12 months at start of study period in Busia program. Non-naïve patients as well as those treated with any other ARV regimen were excluded from the study.

### Ethical approval

This study was approved by the Ethical Review Board of Médecins Sans Frontières and the Kenya National Ethical Review Committee in KEMRI (Kenya Medical Research Institute). Written inform consent was not obtained because the majority of local people participating in this study were illiterate. Therefore, and after a thorough explanation of the study purposes, and in presence of a third person (family related if possible or health care worker instead), we requested to all participants to acknowledge their verbal informed consent. Ethical Review Board of Médecins Sans Frontières as well as Kenya National Ethical Review Committee in KEMRI (Kenya Medical Research Institute) approved this consent procedure.

### Laboratory procedures

On the day of enrolment, a venous blood sample of 10 mL was taken and divided into two parts: one had a CD4 count done at the Busia lab using the FASCOUNT machine using the manufacturer's guidelines, and the other one was sent to Kenya Research Institute Nairobi (KEMRI) in a plasma preparation tube where viral load testing was done using the NucliSENS EasyQ HIV equipment, version 1.2, using the manufacturer's guidelines, with limit of detection of 50 copies/ml.

Patients having a viral load >5.000 copies/ml provided an additional sample for preparation in dried blood spot (DBS) [13], [14]. Even though viral load >1.000 copies/ml would be enough to perform genotyping, we decided to select patients with >5.000 copies/ml for two main reasons: the lower sensitivity of genotype testing using Dried Blood Spots Filter Papers and the fact that due to technical constraints, that is, inability to keep cold chain during the whole transportation process, we used BD Vacutainer PPT Plasma Preparation tubes (PPT) (Becton Dickinson and Company, Franklin Lakes, NJ). Several studies have reported that the use of Vacutainer plasma preparation tubes (PPTs) to transport plasma specimens have consistently yielded higher HIV RNA levels than EDTA tubes [15], [16], [17], [18].

Fifty microlitres of whole blood and plasma were used to make each spot respectively. These were dried overnight on special drying racks, packed and sent to the Research Laboratory from Hospital Universitari MutuaTerrassa, in Barcelona, for HIV resistance genotyping tests. Before study initiation, training was implemented among technicians (local staff) responsible of sampling procedure.

### Self report adherence questionnaire

Adherence was measured using a simple questionnaire translated to the local language. The questions were structured based on the WHO adherence criteria. Pill counts for the patients were done by adherence counsellors.

### Definition of treatment and virological failure

For the purpose of our study we performed a viral load test for all patients that were enrolled in the study. Samples with viral loads ≥5.000 copies/ml were selected for genotype testing.

### Treatment failure

Treatment failure based on CD4 test criteria was defined as either a CD4 below the baseline or less than 50% of peak at 12 months or CD4 <100 cells/mm^3^ after 12 months of therapy. Treatment failure based on clinical criteria was defined as the occurrence of either a new or a recurrent disease defining WHO 3 or stage 4 at 12 months from the start of medication [19].

### Virological failure

Currently, WHO defines virological failure as plasma HIV-1 RNA level >5.000 copies/ml after 6 months of treatment. However, at the time of the study (2008) the programme was following WHO 2006 recommendations where the cut-off for virological failure was >10.000 copies/ml,. Notwithstanding, in our study the aim was to assess the level of HIV drug resistance mutations and to describe and rank specific HIV-Drug Resistance mutations and mutation patterns among patients not achieving virological suppression. Therefore, all patients with VL >5.000 had their sample collected for genotype testing.

### Sample collection and shipment

Patients having a viral load ≥5.000 copies/ml provided an additional blood sample to prepare dried blood (DBS-FP) on Schleicher and Schuell 903 filter paper to send to Hospital Universitari MutuaTerrassa, AIDS research Unit, in Terrassa, for genotyping test. The BD PPT™ tubes (plasma preparation tubes) were used to separate plasma, following the manufacturer's instructions. Briefly, 5 ml of whole blood were collected into the PPT^TM^ tubes using universal phlebotomy practices, and briefly gently inverted to mix. Of this sample, 400 ul were spoted on the filter paper in the 5 pre-marked circles (50 ul each). The remaining sample was centrifuged at 3000 rpm for 10 minutes to obtain plasma. This plasma was collected into vacutainer tubes for viral load assay. Filter papers were leaved to dry on drying racks overnight in a horizontal position at a room temperature. They were then packed in glycine envelopes with desiccants to keep them dry and shipped to the genotyping laboratory in Barcelona following the laid down procedures for sample packaging and shipment. Samples were kept during the whole procedure and transportation at room temperature. In our laboratory, total HIV-1 RNA from DBS was extracted using Nuclisens method (Easymag, Biomerieux). A unique fragment of 1023 bp of pol gene containing the major part of PR and RT was amplified using in-house RT-PCR and nested PCR previously described in the literature [13].

Genotyping was validated using the ViroScore Suite (ABL, Luxembourg, v3.9.2).

### Statistical analysis

Qualitative and quantitative variables were described using percentages and 95% confidence intervals (CI), means and standard deviation or median and interquartile ranges (IQR), depending upon distribution was normal or not. Comparisons between patients with or without virological failure were examined using Pearson's chi square test or Fisher's exact test for qualitative variables and t-test or U Mann-Whitney test for quantitative variables. Previously, Levene test was performed to determine homogeneity of variance.

In addition, proportion comparison test for independent samples through Z statistic were performed for comparison of adherence results.

P values less than 0.05 were considered statistically significant. Analyses were conducted using the statistical package StataSE vs9.0 (Stata Corporation, College Station, Texas, USA).

## Results

**Figure 1:**
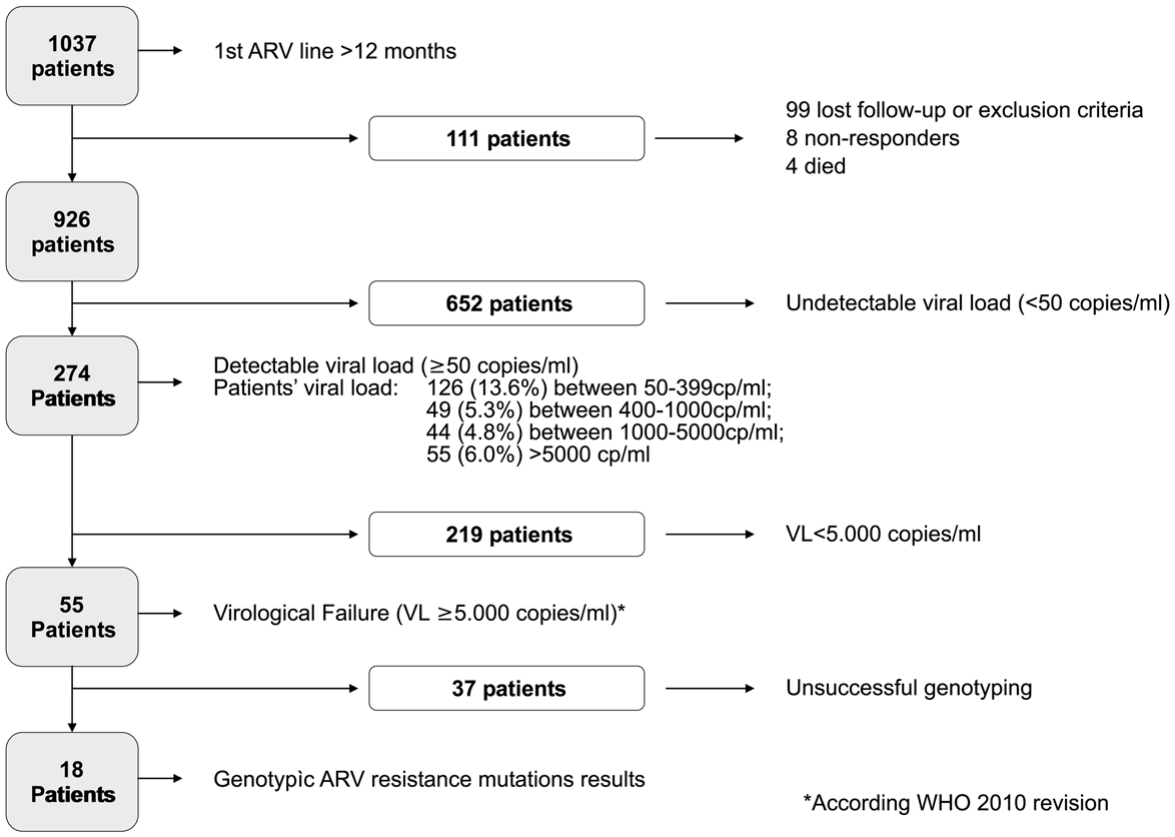
Patient's disposition.

Among 1037 recruited patients under HAART during ≥12 months, 926 were included in the analysis (Figure 1). Out of 111 non-included patients only 8 (7.2%) were non-responders and 4 died, the remaining patients were lost of follow-up, or have an exclusion criteria. Mean time on ARVs were 38.8 months (range: 17.0–60.0) and patients mean age were 42.1 y. (range: 18,6–73,1), with a higher proportion of females (67.3%) (Table 1).

**Table 1:** Patients with VL >5.000 copies/ml (N = 55).

VL >5.000 copies/ml	55/926 (5,9%)
Mean age	37.9 (range:18.7–72.5)
Females	34 (61.8%)
Clinical failure	10 (18.2%)
Median CD4+	116 cells/mm3 (IQR: 54–189)
Median VL (RNA-VIH)	32.000 copies/ml (IQR: 11.000–68.000)
ARV regimen started: d4T/3TC/NVP AZT/3TC/NVP	53 (96.4%)2 (3.6%)
Mean duration on HAART	40.1 months (range: 20–52)

Among the whole population studied, 274 patients (29,7%, 95% CI: 13.6–18.4) had detectable viral load (≥50 copies/ml) and 55 (5.9%, 95% CI: 4.4–7.5) had a viral load ≥5.000 copies/ml, thus underwent genotypic study. Among 55 patients of genotypic study, mean age was 37.9 years (range: 18.7–72.5) and 34 (61.8%) were females. Only 10 out of 55 patients (18.2%) experienced clinical failure, (treatment failure based on clinical criteria) defined as the occurrence of either a new or a recurrent disease defining WHO 3 or stage 4 at 12 months from the start of medication. The median CD4 cell count and HIV RNA were 116 cell/mm3 [interquartile range (IQR) 54–189] and 32,000 copies/ml respectively (IQR: 11.000–68.000) (table 1). ART regimen started at time of inclusion were d4T/3TC/NVP in 53 (96.4%) and AZT/3TC/NVP in 2 patients (3.6%). Regarding mean time on HAART, there were no significant statistical differences between patients that had virological failure (n = 55) and those patients (n = 871) who did not (mean: 40,1 vs. 38,7 months respectively).

Of the 55 samples, only 18 (33%) could be amplified and evaluated by genotyping. Based on Stanford University HIV Drug Resistance Database, we inferred subtypes from entering FASTA sequence information: the majority of samples were subtype A (13/18; 72,2%), subtype D (4/18; 22,2%) and G (1/18; 5,6%).

Among 18 samples sequenced on PR and RT genes, 15 out of 18 (83.3%, 95% CI: 58.6–96.4) showed RT resistance associated mutations (RAMs). The median number of resistance mutations (any class) was 5 (IQR: 2,75–6). Three samples had no mutations identified (table 2).

**Table 2:** RAMS among patients with virological failure (VL>5.000 copies/ml) amplified and evaluated by genotyping.

Resistance Associated Mutations (RAMs)	N (%)
Some RAMs	15/18 (83.3%)
• **TAMS** (M41L, D67N, K70R, V75I, L210W, T215Y/F, Q151M)	11/15 (73.3%)
• **M184V**	15/15 (100%)
• **NNRTI** (K101E, K103N/S, G190A, V108I, Y181C/V, Y188L)	15/15 (100%)

**Figure 2:**
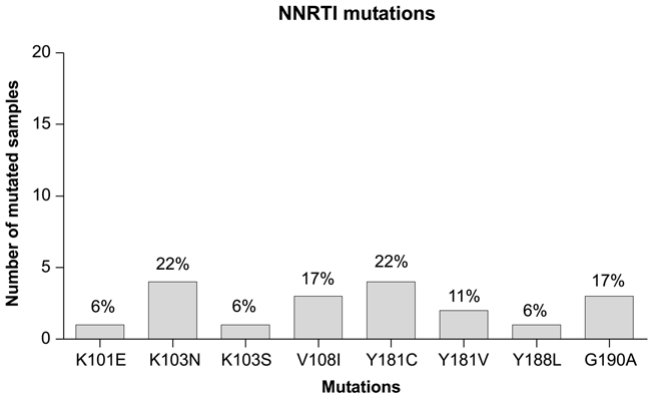
NNRTI's resistance mutations prevalence.

Fifteen out of 18 patients (83.3%) carried NNRTI resistance associated mutations, although there were no patients with only NNRTI mutations. The median number of NNRTI mutations was 1 (IQR: 1–2). The most frequent NNRTI mutations were K103N/S (27.8%), followed by Y181C (22.2%), V108I and G190A (16.7%), Y181V (11.1%), K101E and Y188L (5.6%) (Figure 2).

**Figure 3:**
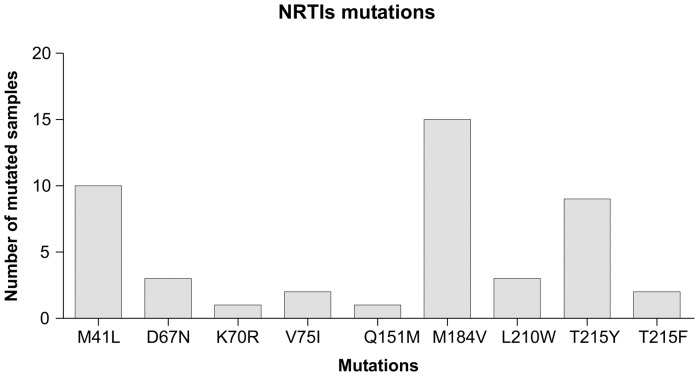
NRTI's resistance mutations prevalence.

Prevalence of NRTI resistance associated mutations are depicted in Figure 3. The M184V mutation was present in virus from 15 patients (83.3%), although never as the only mutation. Four patients (22.2%) carried virus with only M184V and NNRTI mutations (Table 3). The most common mutation pattern was M184V and NNRTI mutations with one or more TAMs, which occurred in 12 (66.7%) patients.

**Table 3:** Genotypic results.

ID	HAART	VL	H*	Protease	Retrotranscriptase
**2**	1	63000	10%	H69K, L89V	**K101E, M184V, G190A, T215Y**
**4**	1	11000	10%	I62V, L63P, I64V, *H69Y*	**K103N, M184V**
**5**	1	5600	10%	M36I, D60E	**M41L**, A98G, **K103N, M184V, T215Y**
**6**	1	5700	10%	H69K, *L89M*	**M41L, V75I**, V90I, **K103N,** *V179I* **, M184V, T215Y**
**8**	1	1000000	10%	*H69Q*, *L89M*	**M41L, V108I**, *V179I*, **Y181C, M184V, L210W, T215Y**
**10**	1	140000	10%	M36I, I62V, *H69R*, *V82Y*, *L89M*	
**11**	1	9200	10%	D60E, H69K, *L89M*	**M41L, M184V, Y188L**, *L210S*, **T215Y**
**13**	1	910000	10%	H69K, *L89M*	*V179I*
**19**	1	6200	60%	*L63Q*, *H69Y*	**K70R, V108I, Y181C, M184V**
**30**	1	29000	30%	M36I, I62V, *L63T*, I64M, H69K, *L89M*	**M41L, V75I**, *V179I*, **Y181C, M184V, T215Y**
**31**	1	47000	30%	M36I, H69K, *L89M*	**M41L**, *V179I*, **M184V, G190A, T215F**
**33**	1	1200000	30%	*E35D*, M36I, H69K, *L89M*	
**36**	1	11000	30%	H69K, *L89M*	**K103S**, *V179I*, **M184V**
**37**	1	130000	30%	L63P, H69K, *L89M*	**M41L, D67N, K103N, Q151M, M184V, T215F**
**38**	1	43000	30%	D60E, H69K, *L89M*	**M41L, D67N**, *V179I*, **Y181V, M184V, L210W, T215Y**
**39**	1	68000	30%	M36I, *L63T*, H69K, V82I, *L89M*, I93L	*K101A*, *V179I*, **M184V, G190A**
**46**	2	170000	30%	H69K, *L89V*, *L90T*	**M41L, D67N**, **Y181V, M184V, L210W, T215Y**
**53**	1	130000	40%	D60E, H69K, *L89M*	**M41L, V108I**, *V179I*, **Y181C, M184V, T215Y**

• **% of humidity.**

• **HAART: 1.-3TC/d4T/NVP; 2.-AZT/3TC/NVP.**

Among patients carrying RAMs, 12/15 (80.0%) harboured RAMs associated to thymidine analogues (TAMs) (M41L, D67N, K70R, V75I, L210W, T215F/Y) (Table 3). All of them (100%) showed also M184V RAM to lamivudine. Of the patients with TAMs containing virus, 16.7% had one, 33.3% had two, and 50.0% had three or more TAMs. The most frequent TAMs were T215F/Y (11/12, 91.7%), M41L (10/12, 83.3%), L210W (3/12, 27,3%), D67N (3/12, 25.0%), K70R (1/12, 8.3%) and V75I (1/12, 8.3%) (Table 3). One patient carried Q151M mutation, associated with M41L, D67N, T215F and M184V, conferring pan-nucleoside resistance, except to tenofovir. There were no patients carrying either K65R or T69 insertion.

In our study all failing patients with HIV-1 RNA >5.000 copies/ml harboured TAMs. Among the three patients having samples with no mutations identified, viral load were high (mean: 750.000 copies/ml; 5,9 logs).

According to Stanford HIV database, 10 out of 15 (66.7%) patients were resistant to both ARV families used, that are stavudine, zidovudine, lamivudine and nevirapine/efavirenz.

Regarding samples sequenced on protease gene, we have found only common polymorphisms not associated with decreased PI susceptibility. We did not find primary RAMs to protease inhibitors (Table 3).

### Adherence results

Adherence questionnaire did not discriminate among those patients harbouring some resistance mutations and those without any resistance at all. In contrast, 30,99% of patients having viral load >5000 copies/ml (n = 55) vs. 44.2% of patients with viral load <5000 copies/ml (n = 871) reported “never missed medications” (p<0,05).

## Discussion

Despite earlier doubts, evidence is available that even in countries with very limited resources, ART programmes based on the public health approach have shown effectiveness equal to that seen in clinical cohorts in USA and Europe using similar regimens [8], [20], [21], [22], [23], [24], [25], [26]. Therefore, fears that HIV/AIDS treatment in RLS would lead to widespread drug resistance have been unfounded. Our study provides additional scientific concerns about comprehensive approach to monitoring, care and treatment management in settings in which resources are limited.

Also virological failure rate in our study is similar than those reported so far in developed countries. Likely, results from several operational research performed in different RLS yielded the same results than our study, with a longer follow-up period (Table 4).

**Table 4:** Virological failure rates over time in different resource limited settings.

Busia Mean Tx duration: 38,8 m. (926 patients)	Malawi^2^ Mean Tx duration: 9 m (397 patients)	Cambodia^3^ Mean Tx duration: 24 m (346 patients)
VL>400 = 16%	VL>400 = 16%	VL>400 = 11.6%
VL>1000 = 10,7%	VL>1000 = 13%	VL>1000 = 9%
VL>30.000 = 3,0%	VL>30.000 = 5.0%	VL>30.000 = 4.3%

2 **Ferradini et al. Lancet 2006; 367:1335–42. ^3^ Ferradini et al. AIDS 2007, 21:2293–2301.**

The ability to collect blood samples on filter paper represents an advantage for HIV drug resistance surveillance and monitoring, particularly in areas that lack the appropriate infrastructure for plasma processing and transport. We used DBS method for genotyping purposes due to its simplicity and the fact that filter paper technology allows shipment with minimal biohazard risks. One of the crucial points for the use of dried fluid spots for drug resistance genotyping in the field is the stability of RNA over time. Dried blood spots can be kept for long periods when refrigerated or frozen in hermetic bags with desiccant but exposure to high temperatures for extended periods seems to be associated with degradation. Few studies have reported on long-term storage or testing under field temperature conditions, and contradictory percentage of genotyping efficiency of blood and plasma sample collection devices such DBS results have been reported, compared with gold standard plasma [27], [28], [29].

In our study, we could amplify to evaluate by genotyping only one third of samples. Such a low percentage of positive genotypic results among failing patients might be explained by different factors, mainly related with the obstinate real life in Operational Research in RLS that make difficult the optimisation of sampling procedure: storage conditions, transportation and cold chain preservation as it was mentioned in methods, in our study samples were kept during the whole procedure and transportation at room temperature. Also, combination of insufficient concentration of viral RNA and decreased quality of RNA in DBS specimens can explain our low PCR amplification rate. Nonetheless, in our study, there were no statistically differences in viral load between amplificated and non amplificated samples. McNulty et al. demonstrated also the same rate of amplification of HIV-1 *pol* in DBS among Cameroonian HIV infected people that were stratified according different plasma viral load (from 5.000 to >50.000 copies/ml) [30]. Moreover, although filter paper were packed in glycine envelopes with desiccants to keep them dry, more than half of the samples (62%) received in our research lab in Terrassa had a humidity degree of 30% or higher, according humidity indicators. We hypothesized that such a high humidity degree was due, at least in part, because Busia OR study was performed during rainy season. Contradictory results in the literature suggest that some degradation of RNA may have occurred during long-term storage at 48°C possibly due to suboptimal storage temperature, humidity or both. High humidity conditions are thought to be detrimental to resistance testing from DBS given the extreme sensitivity of HIV nucleic acids to degradation in the presence of humidity [14].

Although resistance mutation patterns were not available among the whole samples sequenced, genotypic results were concordant with ARV treatment received by failing patients, which is either nevirapine/d4T/3TC or nevirapine/AZT/3TC (Table 3). Resistance mutations profile allowed us to switch ART to a second-line therapy based on a backbone of either tenofovir/FTC or ABC/ddI with a boosted protease inhibitor (lopinavir). The majority of samples amplified from failing patients carried out M184V RAM (high level resistance to 3TC/FTC), NNRTI resistance associated mutations (cross resistance to NVP and EFV), as well as several thymidine analogue resistance mutations (TAMS), conferring different degrees of resistance to NRTIs.

The absence of any relevant resistance mutation among three patients probably corresponds to patients who did not take medication at all, reinforcing the importance of treatment adherence in the context of lack of regular access to VL monitoring and genotype. Intensive therapeutic education, counselling and psychosocial support might explain the very low rate of lost-to follow-up patients observed, thus emphasizing that human resources remain a key factor in resource-limited settings.

The mutations observed were those expected under the first-line regimen used, and the great majority of patients were resistant to nevirapine/efavirenz (100%), lamivudine (100%) and stavudine (83%). Overall, these observations are similar to other first-line cohort studies in RLS describing HIV-1 mutation patterns [20], [31], [32], suggesting that switch after a first-line ART failure in RLS might be feasible without a genotypic test. However, some authors have warned about the fact that d4T-based initial therapy in patients infected with subtype C virus selects for a broader array of mutations, including the K65R mutation, conferring resistance to d4T, ddI, ABC, 3TC, FTC, and TDF [10]. Subtypes profile found in our study are very concordant with results of a multicenter study carried out in four African cities, one of them closer from Busia (subtype distribution in Busia and Kisumu were 72% vs. 71%, 22% vs. 20% and 5,6% vs. 2% for A, D and G respectively) [33].

The accumulation of thymidine analogue and other resistance mutations can confer cross-resistance to many, and possibly, to all NRTIs that might be used in a second line-regimen. This phenomenon may be less likely when tenofovir is included in the first-line regimen. However, despite WHO 2010 recommendations, tenofovir is still commonly unaffordable in RLS. This data, together with our findings showing high TAMS mutation rate in our cohort, as well as the high rate of d4T-related polineuropathy–data not shown-, suggest that in RLS we desperately need other changes to make treatment more comparable to that in industrialised countries. We should consider moving away from d4T, rarely used in the North, to the easier tenofovir, although it would also cost more money (tenofovir currently costs four times more).

UNAIDS 2010 global report have included important key elements to improve the efficiency and quality of HIV treatment and care: a) start ARV therapy earlier, b) use less toxic and more patient-friendly options (fixed dose combinations) and c) use of laboratory monitoring tools such as CD4 and viral load counts. Nevertheless, introduction of safer but currently more expensive first-line ARTs, as well as viral load monitoring, needs to be phased-in as currently they may not be feasible or affordable in many high-burden settings with low coverage, less developed health systems, limited laboratory capacity, finite budgets and competing health priorities.

Newer guidelines [34] suggest also starting one of the following regimens in ART-naïve individuals eligible for treatment: AZT + 3TC + EFV; AZT + 3TC + NVP; TDF + 3TC or FTC + EFV, TDF + 3TC or FTC + NVP. A boosted protease inhibitor (PI/r) plus two nucleoside analogues (ZDV, 3TC and tenofovir as a NRTI backbone) are recommended for second-line ART, being ATV/r and LPV/r the preferred boosted PI's. The main objective of these recommendations try to avoid the disfiguring, unpleasant and potentially life threatening toxicity of d4T, the need to select regimens suitable for use in most patient groups, and the benefits of using fixed dose combinations. Therefore, with the increasing need for availability of second-line regimens, there is a reasonable argument in support of the widespread, rational use of viral load testing. Although still expensive for RLS at present, it has the potential to prevent unnecessary switches to expensive second line therapies.

Although recent modelling study support little added survival benefit to the addition of laboratory viral load monitoring compared with clinical monitoring alone [35], not only early survival need to be considered [36]. The long term consequences of high viral load must be calibrated. High viral load on treatment is associated with a greater probability of developing resistance. Therefore, despite that, according some data, from a survival point of view benefits from viral load monitoring might be modest, the accumulation of resistance mutations will confer difficulties to find a cost-effective subsequent treatment regimens, especially in settings with limited second line options and where people will still need decades of therapy. Moreover, the same modelling strategy authors have demonstrated in a more recent study that to preserve current first line drugs as widespread treatment options for future generations, there is a long term need for introduction of some form of cheap, practical, and sustainable viral load monitoring in resource limited settings which can be used in rural as well as urban settings. Also, their results indicated that even very infrequent (e.g. 3 yearly) testing, is likely to provide significant benefit in reducing resistance transmission [37].

In this regard, recently, some authors have presented data from an operational research trial in Zambia, aiming to ascertain the added value of routine viral load testing. This cluster randomized trial of routine versus discretionary (if clinical failure criteria met) viral load monitoring among adults starting ARV therapy, demonstrated that routine viral load testing clearly limits the amount of time spent on a failing regimen in RLS. Time between virological failure and change of the regimen to second line treatment among routine viral load testing group was 168 days compared with 560 days among control group, thus with substantially less time (>1 year) on a failing regimen and higher rate of change to a second line regimen [38]. Results of the resistance and cost-effective analysis are still pending, and they will provide important information on the costs of that delay for future treatment options.

## Conclusion

We have long known of increased mortality in African patients on ART compared with outcomes elsewhere. A conclusion would seem that if the future is to be different, we have to intervene earlier. Current worldwide expansion of ARV therapy for HIV in resource-limited countries entails new challenges to be considered. More than 6,5 million people are receiving ART, with the majority of patients on treatment worldwide now being from resource limited settings. Important treatment-related issues identified by several scientific communities, such as when to switch therapies, long-term consequences of virological failure after current first ART strategy, toxicity, adherence, drug-drug interactions or optimal treatment of pregnant women, need to be addressed immediately.

Moreover, controversial issues about comprehensive laboratory monitoring such as routine monitoring viral load for safety and efficacy in these settings should be elucidated. VL testing could increase in importance as a guide for clinical decisions on when to switch to second-line treatment and on how to optimize the duration of the first-line treatment regimen, minimizing the impact of ARV resistance mutations on treatment. Therefore, now we must pay attention to monitoring to limit the costs associated with widespread use of expensive second-line therapy, ensuring that VL testing becomes affordable, simple and easy to use in RLS.
